# The relationship between circulating concentrations of C-reactive protein, inflammatory cytokines and cytokine receptors in patients with non-small-cell lung cancer

**DOI:** 10.1038/sj.bjc.6602248

**Published:** 2004-11-30

**Authors:** D J McKeown, D J F Brown, A Kelly, A M Wallace, D C McMillan

**Affiliations:** 1 University Department of Surgery, Royal Infirmary, Glasgow G31 2ER, UK; 2 Department of Clinical Biochemistry, Royal Infirmary, Glasgow, UK

**Keywords:** inflammatory cytokines, cytokine receptors, C-reactive protein, non-small-cell lung cancer

## Abstract

The relationship between circulating C-reactive protein concentrations and potential cytokine and receptor mediators (interleukin-6, leukaemia inhibitory factor (LIF), ciliary neurotrophic factor (CNTF), soluble IL-6 receptor, soluble gp130, soluble TNF receptor, interleukin-1 receptor antagonist and interleukin-8 (IL-8)) of this acute phase protein were examined in healthy subjects (*n*=11) and patients with non-small-cell lung cancer (*n*=50). Leukaemia inhibitory factor and CNTF were below detection limits in all controls and patients. C-reactive protein, interleukin-6, soluble gp130, soluble TNF receptor, interleukin-1 receptor antagonist and IL-8 concentrations were significantly elevated in cancer patients (*P*⩽0.001). Cancer patients with elevated C-reactive protein concentrations had greater concentrations of interleukin-6 (*P*<0.01) and interleukin-1 receptor antagonist (*P*<0.05). On regression analysis only interleukin-6 was independently associated with C-reactive protein (*r*=0.616, *P*<0.001). Interleukin-6 is an important independent mediator of elevated C-reactive protein concentrations in patients with non-small-cell lung cancer.

Non-small-cell lung cancer (NSCLC) is the most common cause of cancer death in North America and Western Europe. Each year in the United Kingdom there are almost 39 000 new cases registered; only 7% are alive at 5 years (Cancerstats, 2004; http://www.cancerresearchuk.org). Most patients present with advanced inoperable disease, few are amenable to surgery and the results of radiotherapy and chemotherapy are poor.

It has recently become clear that the systemic inflammatory response, as evidenced by elevated circulating concentrations of C-reactive protein, is an important factor in the progressive nutritional decline of these patients and is a prognostic factor independent of stage, performance status and treatment (Scott *et al*, 2002; Forrest *et al*, 2003, 2004).

In the NSCLC patient the basis of the elevated C-reactive protein concentrations is unclear. Previous work has shown a strong positive association between interleukin-6 (IL-6) and C-reactive protein concentrations (Yanagawa *et al*, 1995; Scott *et al*, 1996). A number of other cytokines, which share structural homology and signal through the gp130-Jak-STAT pathway, have, however, been implicated in the elaboration of acute phase proteins in cancer (Heinrich *et al*, 1998). For example, *in vitro* studies have shown that leukaemia inhibitory factor (LIF) and ciliary neurotrophic factor (CNTF) stimulate increased acute phase protein production (Baumann *et al*, 1993). Additionally, there is evidence that soluble receptor subunits involved in IL-6 signal transduction, the soluble IL-6 receptor (sIL-6R) and the soluble gp130 receptor (sgp130), may also be important in regulating IL-6 activity and the acute phase protein response (Jones and Rose-John, 2002). Also, soluble TNF receptor (sTNF-RII), IL-1 receptor antagonist (IL-1Ra) and interleukin-8 (IL-8) have been reported to be associated with the systemic inflammatory response in lung cancer patients (Yanagawa *et al*, 1996; Simons *et al*, 1999; Alexandrakis *et al*, 2000).

The aim of the present study was to examine the relationship of the inflammatory cytokines, cytokine receptors and circulating concentrations of C-reactive protein in patients with NSCLC.

## PATIENTS AND METHODS

### Patients

Patients with a firm clinical or histological diagnosis of locally advanced or metastatic advanced NSCLC from palliative care centres in the West of Scotland were studied. Healthy subjects individuals were recruited from volunteers of the palliative care centres to serve as normal controls. The study was approved by the local research and ethics committee. All patients and controls were informed of the purpose of the study prior to entry and gave written, informed consent.

### Experimental design

A blood sample was collected for routine laboratory analysis of white cell count, albumin and C-reactive protein. The minimum detectable concentrations for C-reactive protein were 6 mg l^−1^. The inter- and intra-assay variability of white cell count, albumin and C-reactive, protein were less than 10%.

A further blood sample was taken and centrifuged and the serum stored at −20°C prior to analysis of inflammatory cytokines and receptors. Circulating concentrations of the proinflammatory cytokines IL-6, LIF, CNTF and IL-8 and soluble receptors sIL-6R, sgp-130, IL-1Ra and sTNF-RII were measured using an enzyme linked immunosorbent assay (ELISA) technique. All samples were measured in duplicate and the mean value used. The minimum detectable concentrations were 2 pg ml^−1^ for IL-6, 6.5 pg ml^−1^ for sIL-6R, 8 ng ml^−1^ for sgp130, 1 pg ml^−1^ for sTNF RII, 14 pg ml^−1^ for IL-1 Ra, 10 pg ml^−1^ for IL-8, 8 pg ml^−1^ for LIF and CNTF (Quantikine ELISA, R&D Systems Europe Ltd, Abingdon, UK). Inter- and intra-assay variability were less than 10% for all assays used.

### Statistical analysis

Data are presented as median and range. Inflammatory cytokine and cytokine receptor concentrations below the threshold of sensitivity of the respective assays were expressed as equal to this threshold. Where appropriate, data were tested for statistical significance using the Mann–Whitney *U* test. As the distribution of C-reactive protein and the inflammatory cytokines and cytokine receptor concentrations were skewed, they were logarithmically transformed prior to stepwise multiple regression analysis for the examination of independent associations with C-reactive protein (SPSS version 11 Inc., Chicago, IL, USA).

## RESULTS

The characteristics of the healthy controls and the NSCLC patients are shown in Table 1

**Table 1:** Characteristics of healthy subjects and patients with NSCLC

	**Healthy controls (*n*=11)**	**NSCLC patients (*n*=50)**	***P*-value**
Age	66 (46–74)	64 (43–83)	0.894
Sex (M/F)	5/6	24/26	0.917
BMI	25.4 (22.4–28.9)	21.9 (14.8–64.2)	0.017
Albumin (g l^−1^)	43 (41–46)	40 (28–44)	<0.001
White cell count (10^9^ l^−1^)	6.7 (4.1–8.4)	9.21 (5.1–23.5)	<0.001
C-reactive protein (mg l^−1^)	<6 (<6–<6)	26 (6–207)	<0.001
			
IL-6 (pg ml^−1^)	<2 (<2–<2)	13 (<2–103)	<0.001
SIL-6R (ng ml^−1^)	16.1 (14.9–24.2)	18.8 (7.8–46.0)	0.291
Sgp130 (ng ml^−1^)	234 (175–290)	298 (227–448)	<0.001
sTNF-RII (ng ml^−1^)	1.5 (1.2–7.5)	2.9 (1.5–6.8)	0.001
IL-1Ra (pg ml^−1^)	286 (129–368)	539 (132–2129)	<0.001
IL-8 (pg ml^−1^)	<10 (<10–<10)	13.9 (<10–118)	<0.001

Median (range).

. Age and sex were similar in healthy subjects and patients. BMI and albumin were lower (*P*<0.05) and white cell count (*P*<0.001) and C-reactive protein (*P*<0.001) were higher in cancer patients compared with controls. Circulating concentrations of CNTF and LIF were less than the detection limit of the assay in all healthy subjects and cancer patients. Circulating concentrations of IL-6, sgp130, sTNF-RII, IL-1Ra and IL-8 were all significantly elevated in cancer patients compared with controls (*P*<0.01).

NSCLC patients were grouped according to whether or not they had evidence of a systemic inflammatory response (C-reactive protein >10 mg l^−1^, Table 2

**Table 2:** Characteristics of inflammatory and noninflammatory patients with NSCLC

	**C-reactive protein ⩽10 mg l^−1^ (*n*=14)**	**C-reactive protein >10 mg l^−1^ (*n*=36)**	
	**Median <6 mg l^−1^**	**Median 58 mg l^−1^**	***P*-value**
Age	61 (44–74)	66 (48–88)	0.119
Sex (M/F)	5/9	19/17	0.278
BMI	23.3 (19.7–31.0)	21.9 (14.8–64.2)	0.287
Albumin (g l^−1^)	42 (38–47)	40 (28–46)	0.014
White cell count (10^9^ l^−1^)	8.50 (5.60–18.87)	9.55 (5.10–23.50)	0.305
			
IL-6 (pg ml^−1^)	2.9 (2.0–22.2)	18.5 (2.0–103.0)	0.001
SIL-6R (ng ml^−1^)	23.3 (14.0–30.7)	18.8 (7.8–46.0)	0.216
Sgp130 (ng ml^−1^)	280 (218–330)	298 (227–448)	0.572
sTNF-RII (ng ml^−1^)	2.5 (1.5–5.1)	3.2 (1.5–6.8)	0.122
IL-1Ra (pg ml^−1^)	378 (234–902)	724 (132–2128)	0.016
IL-8 (pg ml^−1^)	<10.0 (<10.0–90.6)	13.9 (<10.0–118.0)	0.152

Median (range).

). The groups were similar in terms of age, sex, BMI, white cell count and circulating concentrations of sIL-6, sgp130, sTNF-RII, and IL-8. In the inflammatory group albumin concentrations were lower (*P*<0.05) and IL-6 (*P*<0.01) and IL-1Ra (*P*<0.05) concentrations were higher. In the cancer patients neither sIL-6R nor sgp130 concentrations were significantly associated with either IL-6 or C-reactive protein concentrations. In contrast, IL-6 concentrations were correlated with the white cell count (*r*=0.294, *P*=0.038).

In the cancer patients who had detectable C-reactive protein concentrations (*n*=41), the relationship between log_10_ C-reactive protein, log_10_IL-6, and log_10_IL-1Ra was examined in multiple linear regression analysis. Only IL-6 was independently associated with C-reactive protein (*r*=0.616, *P*<0.001).

## DISCUSSION

In the present study neither of the IL-6 type cytokines LIF and CNTF were detected in the sera of normal healthy controls or patients with NSCLC. Although LIF and CNTF have been shown in rodent studies to induce an acute phase protein response and weight loss (Mori *et al*, 1991; Xu *et al*, 1998) our results are consistent with the largely undetectable levels reported in patients with rheumatoid arthritis (Okamoto *et al*, 1997). We cannot preclude, however, the possibility that these cytokines may be active systemically at concentrations below the sensitivity limit of current assays.

It has been proposed that soluble receptor subunits may be important in regulating IL-6 activity and C-reactive protein production (Jones and Rose-John, 2002). In the present study, however, neither sIL-6R nor sgp130concentrations were significantly different between the inflammatory and noninflammatory cancer patients. Furthermore, neither sIL-6R nor sgp130 concentrations were significantly associated with either IL-6 or C-reactive protein concentrations in the cancer patients. Although measurement of soluble forms of interleukin-6 receptor and gp130 provide a total evaluation of the active concentrations of these mediators, both soluble receptor components exist in several forms and the ELISA determinations do not preclude specific changes in individual sgp130 or sIL-6R isoforms.

In the present study only IL-6 and IL-1Ra were significantly associated with C-reactive protein concentrations in patients with NSCLC. By multiple regression analysis, however, only IL-6 was independently associated with C-reactive protein concentrations. The results of the present cross-sectional study do not preclude the relationship between interleukin-6 and C-reactive protein simply reflecting the simultaneous release of these factors. Nevertheless, taken together these results would suggest interdependence and that C-reactive protein, a routinely available and well-standardised clinical laboratory measurement, is a useful surrogate measure of interleukin-6 activity in patients with NSCLC.

The source of IL-6 in patients with NSCLC remains uncertain since both a variety of host cells and the tumour cells have been shown to produce IL-6. In the present study, IL-6 was only weekly correlated with white cell count in the patients with NSCLC. This may suggest additional sources of IL-6 in these patients. For, example, some lung cancer cell lines have been shown to produce IL-6. Indeed, there is some evidence that IL-6 produced by such tumour cells may act as an autocrine growth factor promoting the survival and invasion of the tumour into surrounding tissue (To *et al*, 2002).

To date, there have been few attempts to moderate the systemic inflammatory response in patients with cancer. Previous work has used a broad-brush approach with nonspecific anti-inflammatory agents (Lundholm *et al*, 1994; McMillan *et al*, 1999). The value of the present study is that it clearly identifies IL-6 from other candidate mediators of the increase in C-reactive protein concentration in a homogenuous cohort of cancer patients.

It also suggests that specific targeting of IL-6 signalling may be appropriate in patients with NSCLC. It is therefore of interest that anti-interleukin-6 receptor antibody has been shown to be clinically beneficial for the treatment of arthritis and Crohn's disease and its action is associated with the suppression of C-reactive protein (Nishimoto *et al*, 2004; Ito *et al*, 2004).

In summary, interleukin-6 is an important independent mediator of elevated C-reactive protein concentrations in patients with NSCLC.
